# Vertical Transposition of the Horizontal Rectus Muscles Combined With the Modified Kestenbaum Procedure for Correcting Abnormal Head Posture Due to Infantile Nystagmus Syndrome

**DOI:** 10.7759/cureus.72602

**Published:** 2024-10-29

**Authors:** Yuki Hayashi, Rie Ichikawa, Masao Takahashi, Keisuke Oba

**Affiliations:** 1 Department of Ophthalmology, Kochi Health Sciences Center, Kochi, JPN

**Keywords:** abnormal head posture, horizontal rectus muscle, infantile nystagmus syndrome, modified kestenbaum procedure, vertical transposition

## Abstract

We report a case of an infantile nystagmus syndrome (INS) with abnormal head posture (AHP) of head tilt and face turn treated with the modified Kestenbaum procedure and vertical transposition of the horizontal rectus muscles. A 23-year-old male patient with a history of eye shaking since early infancy presented to our hospital for correction of AHP. He had right-beating jerk nystagmus in both eyes, 15° right head tilt, and 25° right face turn. Given that the patient’s nystagmus worsens with left head tilt and left face turn, this reinforces the diagnosis of AHP due to INS. We performed vertical transposition of the horizontal rectus muscles combined with the modified Kestenbaum procedure, that is, right lateral rectus muscle (LR) resection 9 mm + 1 muscle width (MW) upshift, right medial rectus muscle (MR) recession 6 mm + 1 MW downshift, left MR resection 7 mm + 1 MW upshift, and left LR recession 8 mm + 1 MW downshift. Postoperatively, a correction of 12° in head tilt and 30° in face turn significantly improved the patient’s AHP. The modified Kestenbaum procedure and vertical transposition of the horizontal rectus muscles effectively corrected the AHP of head tilt and face turn due to INS.

## Introduction

Infantile nystagmus syndrome (INS) is an ocular motor disorder that presents in early infancy. In the early months after onset, the nystagmus is predominantly pendular, which shifts to jerk nystagmus by 18 months of age, allowing for better visual acuity through improved foveation [1]. INS is usually associated with a null zone, a specific position of the eyes where the nystagmus is least intense. If the null zone does not align with the primary gaze position, the patients adopt an abnormal head posture (AHP) to reduce the intensity of the nystagmus. The most common AHP is a face turn, followed by chin up or down positions, and the least common is a head tilt [2]. Extraocular muscle surgery for correcting a single AHP, such as a face turn, is well established [3-5]. However, in the literature, there are only a few case reports on the surgery for complex AHPs in INS, and a standardized approach has not been established [2,6,7].

Therefore, we report a case of a patient with INS and a complex AHP of head tilt and face turn, which were successfully corrected using the modified Kestenbaum procedure and vertical transposition of the horizontal rectus muscles.

## Case presentation

The patient was a 23-year-old man, whose main complaint was a head tilt and face turn. There was no medical or family history to note. He had nystagmus since early infancy, but there were no organic abnormalities in the fundus, and his visual acuity development was good. At the age of nine years, he presented to our hospital with horizontal nystagmus, right face turn, and right head tilt. The nystagmus increased with rightward vision and reduced with leftward and near visions and was diagnosed as a compensatory head posture from INS. The patient did not wish to undergo surgery and had not seen an ophthalmologist for a while, but at the age of 23 years, he requested AHP correction and was referred to our hospital by a nearby eye clinic.

**Figure 1:**
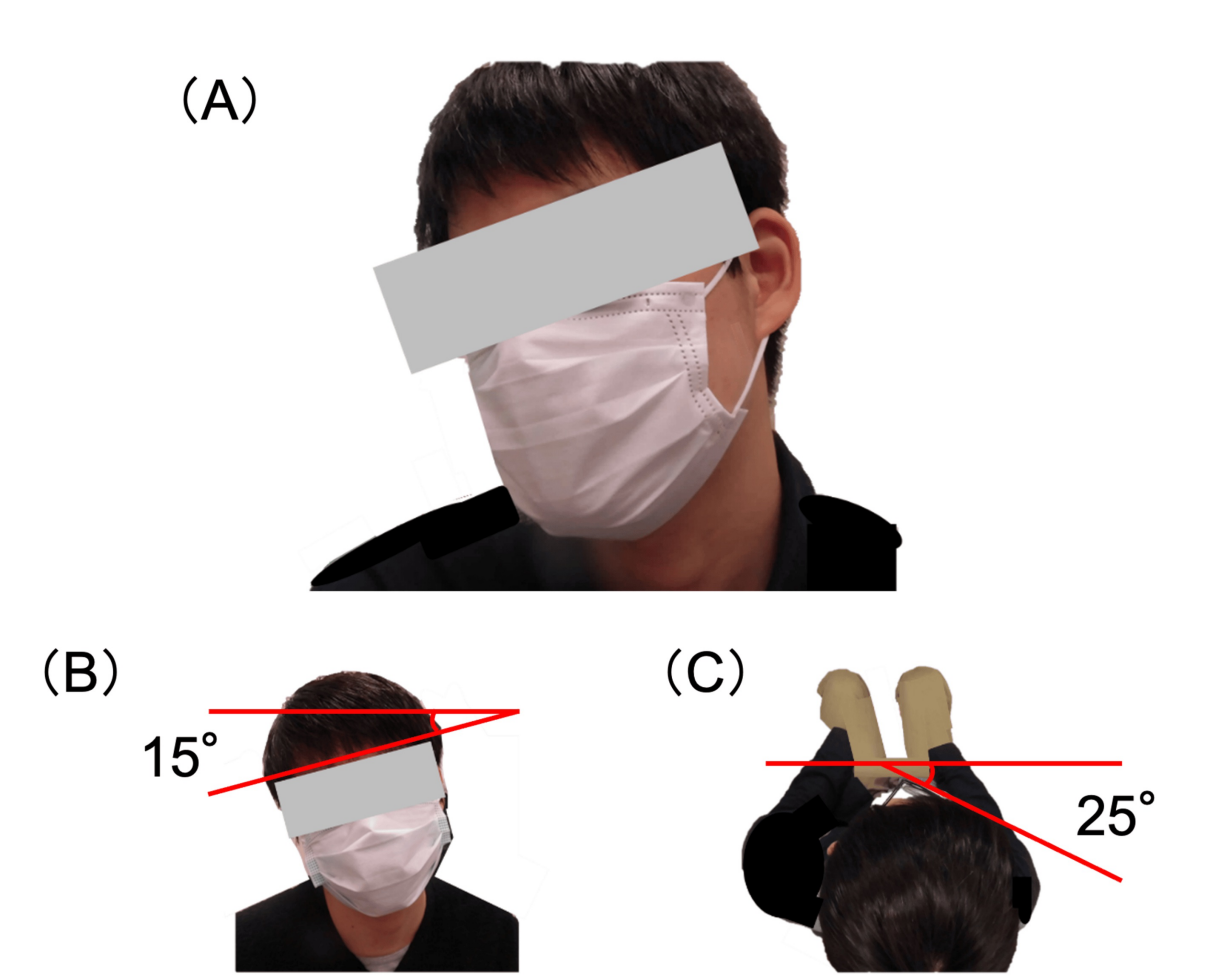
Preoperative head posture. (A) Right face turn and head tilt are mixed. (B) Right head tilt of 15°. (C) Right face turn of 25° (overhead view).

On initial examination, visual acuity was 0.15 (0.7 -1.0D = C-1.0D A 10°) in the right eye and 0.04 (0.8 -5.5D = C-2.0D A 15°) in the left eye, and intraocular pressure was 16 mmHg in the right eye and 17 mmHg in the left eye. The eye position was orthotropic, and the Bielschowsky head tilt test was negative. He had right-beating jerk nystagmus in both eyes, 15° right head tilt, and 25° right face turn (Figure 1).

The nystagmus increased with left head tilt and left face turn. There were no abnormalities in the anterior segment, ocular media, or fundus.

**Figure 2:**
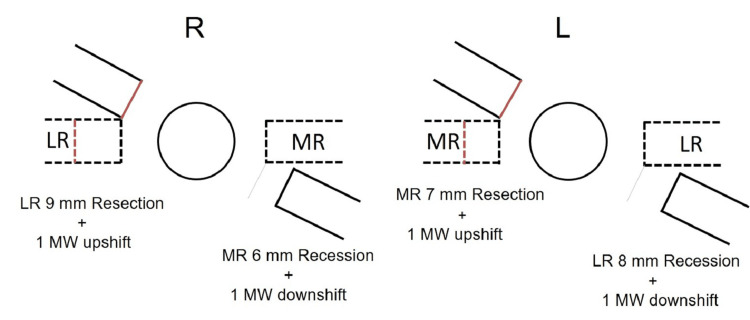
Schema of the surgery drawn up by the authors. Modified Kestenbaum procedure to correct right face turn, combined with upshift of right LR and left MR and downshift of right MR and left LR to correct right head tilt. LR: lateral rectus muscle, MR: medial rectus muscle, MW: muscle width.

To correct the face turn and the head tilt, we performed the modified Kestenbaum procedure combined with vertical transposition of the horizontal rectus muscles under general anesthesia, that is, right lateral rectus muscle (LR) resection 9 mm + 1 muscle width (MW) upshift, right medial rectus muscle (MR) recession 6 mm + 1 MW downshift, left MR resection 7 mm + 1 MW upshift, and left LR recession 8 mm + 1 MW downshift (Figure 2).

**Figure 3:**
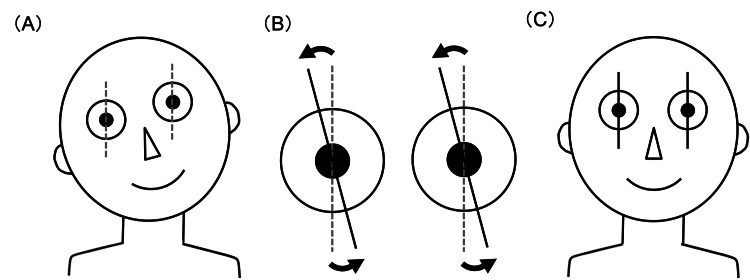
Surgical concept for correcting head tilt. (A) Right head tilt. (B) The right eye was exocyclo-rotated and the left eye was incyclo-rotated. (C) Head tilt was corrected. Both eyes were surgically rotated in the direction of the head tilt. Adapted from reference [11] (Creative Commons Attribution-NonCommercial-ShareAlike License (CC BY-NC-SA).

All vertical transpositions were performed parallel to the spiral of Tillaux. By applying vertical transposition of the horizontal rectus muscles, the right eye was exocyclo-rotated, and the left eye was incyclo-rotated (Figure 3).

**Figure 4:**
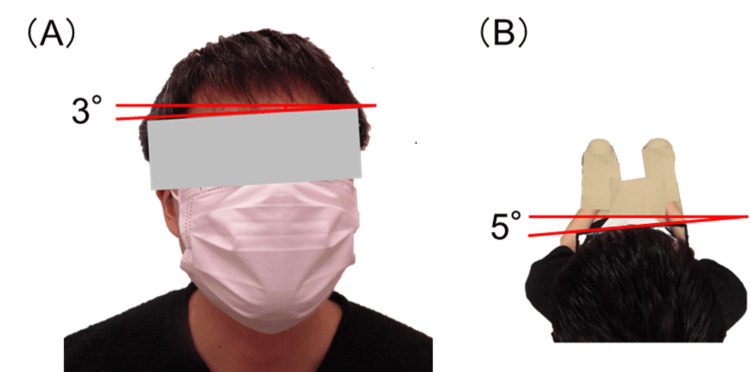
Postoperative head posture. (A) Right head tilt was reduced to 3°. (B) Right face turn was slightly overcorrected, 5° to the left (overhead view).

One month postoperatively, the right head tilt was reduced to 3°, and the right face turn was slightly overcorrected (5° to the left), which significantly improved the AHP (Figure 4).

The corrections were 12° in head tilt and 30° in face turn. All extraocular movements were normal. There was no postoperative complication, such as diplopia or cyclovertical strabismus.　　

## Discussion

The principle involved in the management of AHP in INS is to shift the eyes to the direction of the AHP, which brings the null zone into the primary position. A face turn is the most common type of AHP, and several surgical approaches have been advocated. The most common techniques include recession of the bilateral yoke muscles (Anderson procedure), resection of the antagonist muscles (Goto procedure), and combination recession-resection of four horizontal muscles (Kestenbaum procedure) [3-5]. The Kestenbaum procedure was modified by Parks, which has been well known as the “5, 6, 7, 8” procedure [5]. Park's modified Kestenbaum procedure is based on the idea that the effect of a recession and resection may differ, depending on whether it is performed on the MR or LR. We prefer the modified Kestenbaum procedure, which has a greater corrective effect than the Anderson procedure. In the present case, the face turn was relatively large at 25°; we added 1 mm to the above procedure: “6, 7, 8, 9” mm. Postoperatively, the face turn was slightly overcorrected by 5°. Considering the possibility of reoccurring after a long period, the surgical result was good. 

The surgical principle in the horizontal plane, to shift the eyes to the direction of the AHP, can also be applied to correct head tilt in the torsional plane. Conrad and de Decker proposed rotating both eyes around the sagittal axis toward the head tilt [8]. If a patient has a right head tilt, the right eye should be exocyclo-rotated, and the left eye incyclo-rotated to shift the null zone to the primary position (Fig. 3). Their proposed procedure, to strengthen and weaken the anterior portion of both oblique muscles, is technically demanding and can cause a hypertropia [8,9]. As a simple approach, de Decker suggested the vertical transposition of the horizontal rectus muscles [10]. Kekunnaya and Jain reported three patients treated with vertical transposition of the horizontal rectus muscles, with improvements of 22°-27° in head tilt [11]. As another method of a similar mechanism, von Noorden et al. reported horizontal transposition of the vertical rectus muscles, with improvements of 20°-25° in head tilt [12]. 

We chose the vertical transposition of the horizontal rectus muscles for two reasons. First, associated face turns can be managed by operating the same horizontal muscles. Second, the horizontal rectus muscles can be easily approached, as compared to the vertical and oblique muscles. Transposition of the horizontal rectus muscles is used in treating several types of strabismus, including A-V pattern strabismus, small hypertropia/hypotropia associated with horizontal strabismus, and cyclotropia [13]. Exposure and surgical techniques are basically the same with these procedures; however, the degree of transposition can vary. The treatment guide indicated by the Nystagmus UK Eye research group (Nuke) recommended 1/2 MW vertical transposition to correct a head tilt of 15° or less and 1 MW vertical transposition for a head tilt of 15° or greater [14]. Since the space between the extraocular muscles is 1 MW, 1 MW is considered the maximum amount of muscle transposition. We performed 1 MW vertical transposition of the horizontal rectus muscles to obtain the maximum corrective effect, with improvements of 12° in head tilt.

AHP consists of three dimensions: horizontal (face turn), vertical (chin up or down), and torsional (head tilt) [15]. If the patients are properly evaluated, a mixed type is the most frequent [2,15]. In most cases, only one dimension, such as the face turn alone, is clinically prominent and needs to be corrected. However, if two or three dimensions are prominent, a combination of surgery for each dimension is required. Spielmann reported a case of a head turn with head tilt treated with a horizontal Kestenbaum procedure associated with a downward transposition of the yoke rectus muscles contralateral to the head tilt [2]. Ganesh et al. reported a case of a triplanar AHP treated with the Anderson procedure for face turn and bilateral superior rectus recession for chin down, adding transposition of the horizontal rectus muscles vertically and superior rectus muscles horizontally in both eyes to correct head tilt [6]. Baldev et al. described almost the same approach that we did, with a correction of 30° for face turn and 20° for head tilt [7].

Although the surgical effect has been demonstrated, the mechanism for the improvement of AHP, especially head tilt, remains unknown. Proponents of this procedure assumed that torsional rotation of the eyes induces a tilt of the visual world; to offset this tilt, the patient is forced to straighten the head [8,12]. Our case showed an improvement of 12° in head tilt, which indicates that this mechanism is working. However, in the cases they reported, where the head tilt was reduced by large amounts (25° to 30° or more) [8], the pure mechanical effect of the surgical rotation of the eyes may not fully explain the improvement. Ohtsuki et al. reported five cases of head tilt that underwent oblique muscle surgery and evaluated the relationship between the reduction degree of head tilt and surgically induced cyclotorsion in fundus photographs [9]. Their data showed that surgically induced cyclotorsion (5°-9°) was less than the reduction degree of head tilt (0°-30°) [9]. In our case, fundus photographs were not taken accurately and therefore could not be assessed.

Brodsky and Holmes hypothesized that surgical rotation of both eyes might realign the vertical orientation to objects in the visual world, obviating the need for a head tilt [16]. AHP associated with INS may be due to a complex combination of visual, vestibular, spino-muscular, and central neurological factors [15]. Visual, vestibular, and somatosensory systems are the main sensory systems for gravitational perception and postural control. The strength of the information varies with its reliability or reality, and their contributions to the gravitational perception are determined by the strength of each information [17]. The surgical rotation of both eyes reduces head tilt, decreasing the difference between visual tilt and the actual direction of gravity, which improves the reliability of visual information and might realign the head position closer to straight.

The binocular rotating technique has broader implications for other AHP cases beyond INS. Türkçüoğlu et al. reported a case of a horizontal gaze deviation and face turn due to a pontine hemorrhage, in which the modified Kestenbaum procedure eliminated the face turn [18]. Brodsky and Holmes reported a case of a patient with lateropulsion (strong feeling of being pulled to one side) and head tilt caused by a partial ocular tilt reaction, where horizontal transposition of the vertical rectus muscles of both eyes significantly reduced head tilt from 45° to 5° [16]. If a patient has a face turn due to a horizontal gaze restriction and head tilt arising from a tilted sensation with cerebrovascular disease, the vertical transposition of the horizontal rectus muscles combined with the modified Kestenbaum procedure that we presented may be considered.

## Conclusions

Our case demonstrated that both head tilt and face turn can be effectively corrected through a simultaneous recession-resection procedure combined with the vertical transposition of the horizontal rectus muscles. One significant advantage of vertical transposition of the horizontal rectus muscles for head tilt is its simplicity, allowing it to be easily combined with the horizontal Kestenbaum procedure for addressing concurrent face turns. In addition, this technique has potential applicability to other AHP cases beyond INS. It is crucial to accurately evaluate the type of AHP and select an appropriate treatment method.
